# Exercise Biomechanics for Health: Evaluating Lifelong Activities for Well-Being

**DOI:** 10.3390/healthcare11060900

**Published:** 2023-03-21

**Authors:** Pedro Forte, José E. Teixeira

**Affiliations:** 1 CI-ISCE, Higher Institute of Educational Sciences of the Douro, 4560-708 Penafiel, Portugal; 2 Department of Sport Sciences, Polytechnic of Bragança, 5300-253 Bragança, Portugal; 3 Research Centre in Sports Sciences, Health, and Human Development, 6201-001 Covilhã, Portugal; 4 Department of Sport Sciences, Polytechnic of Guarda, 6300-559 Guarda, Portugal

**Keywords:** biomechanics, load, lifelong, performance, health

## 1. Exercise Biomechanics for Health

Biomechanics is a multidisciplinary study of the mechanical laws and principles that govern human movement and the functioning of biological systems [1]. In the context of physical activity, exercise and physical fitness, biomechanics plays a crucial role in understanding the most efficient and effective ways to improve health and well-being. In addition, biomechanics aim to improve athletes’ performance as well as the movement efficiency of the whole population, leading to a better quality of life. To that end, concerns about optimal economy of movement, strength, power, stability, postural alignment, range of motion and athletic performance are some of the most important topics to address in the study of exercise biomechanics [2].

By studying optimal movement patterns, we aim to understand proper movement patterns and techniques, with the objective to reduce the risk of injury and improve exercise efficiency, thus leading to better health outcomes [3]. Optimal movement refers to the most efficient and effective ways to perform a specific exercise or movement in terms of reducing the risk of injury and illness, as well as improving exercise efficiency, thus leading to better health outcomes [4]. In this field of research, body alignment, joint angles, muscle activation patterns and the transfer of force through the body are variables to consider when assessing optimal movement. For this purpose, specialists (healthcare technicians and medical doctors, including physiotherapists, rehabilitation nurses and medical doctors in sports medicine and physiatry; as well as fitness technicians, including exercise physiologists, fitness instructors and strength and conditioning coaches) may use this information to design and apply evidence-based exercises in training and technician programs.

Exercise biomechanics constantly deal with strength levels, stability and postural alignment. Physical exercise can improve strength and stability, whereby specialists may help individuals to understand and correct posture and misalignments and control strength and stability levels, thus contributing to reducing the risk of injury and illness [5]. Postural imbalances are usually related to stress on the joints and muscles, leading to pain, discomfort and functional decline. Thus, proper alignment of the joints during exercise is crucial to reduce the risk of injury and improve overall function and health. In addition, improved flexibility and range of motion is another topic of relevance in this field; specialists can design different stretching exercises to improve flexibility that target specific areas of the body [6,7]. This may reduce pain and musculoskeletal disorders and, thus, improve overall health and quality of life. Concerning optimal athletic performance, technique efficiency is paramount during strength and power training exercises, as well as during sports-specific sports-based movements [8,9]. This helps to minimize the risk of injury and ensures the efficient use of energy and muscular contribution, leading to improved performance (lower cost for better performance). The biomechanical kinetic and kinematic parameters will help with design programs and allow them to increase power and explosiveness, and, thus, to enhance coordination, reaction time and overall performance.

Proper exercise biomechanics involves the correct form and technique in different joint and anatomical planes during physical activity, exercise and sports activities to ensure that the joints, muscles and bones are moving in a safe and efficient manner. In terms of health and wellness, where physical activity plays an important role, different types of physical activity may require different biomechanical techniques as well as physiological and physical demands. Thus, a healthcare professional or trained fitness professional should be consulted before starting a new exercise program for assessment, prescription and control. The biomechanics of the exercise provide a better understanding of distinct physical limitations, allowing individuals to adjust their exercise program accordingly. The literature continues to call for exercise biomechanics research related to health. Thus, a call for research that emphasizes the importance of methods and information to improve the practices of coaches and players remains important [10,11].

Contributions that, from a biomechanical viewpoint, will explain and examine several biomechanics-related topics in exercise and sports. Discussion of useful applications and/or many other topics of interest has been reported; physical activity, rehabilitation, fitness and competitive sport are appreciated by the exercise biomechanics research community. For that, testing methods, equipment and evaluation techniques will allow us to improve the knowledge required to monitor and evaluate the biomechanical load in health-related physical activity, exercise and sports practice.

## 2. Lifelong Biomechanics

Lifelong biomechanical kinetic and kinematic analyses are the focus of studies on human movement patterns and forces, as well as their repercussions on the body (physical and physiological impact) throughout time [9]. By analyzing these patterns and the mechanics of human movement, individuals can better understand how their physical activity and exercise habits affect their health and physical condition, as well as how they contribute to general well-being [12]. Biomechanical overload can lead to overuse injuries, joint degeneration and other chronic conditions. On the other hand, a lack of biomechanical load can lead to decreased strength, balance, flexibility and physical function. To assess the effects of biomechanical load on health, it is important to consider a variety of factors, especially physical and exercise methodology principles such as intensity, duration and frequency. Additionally, the individual’s age, health status and physical abilities should be also considered in biomechanics [13,14]. By taking these factors into consideration, individuals can better understand the effects of their physical activity and exercise on their body over time and make informed decisions about their exercise program to optimize their physical fitness, health and well-being.

Tools and technologies will help researchers and practitioners to better understand the mechanics of human movement, and can be used to improve exercise programs, injury prevention strategies and overall athletic performance. Testing equipment, instruments, gears and software used in the field of exercise biomechanics will allow researchers to assess and analyze human movement. Some of the most commonly used techniques are motion capture systems, which analyze the movement of joints, bones and muscles and can be used with sensors, cameras or wearable devices [9]. Among the various instruments, force and pressure plates are used to measure the force and power generated during movement as well as the ground reaction forces and pressure distributions during the movement [15]. Electromyography (EMG) is used to measure muscle activation and electrical activity during movement [16]; goniometers and inclinometers are used to assess joint angles/range of motion [16]; the simulation software allows for evaluation of human movement in a virtual environment, considering kinetic and kinematic variables in assumed conditions (controlling inputs) [17]; and, finally, wearable devices, such as accelerometers and gyroscopes, are used to monitor and record movement data in real time [18]. Along these lines, additional research is required in order to test more recent and different methodologies to assess the lifelong biomechanical load, as technology, information and data are becoming increasingly fast, user-friendly and accessible.

## 3. A Call for Research Topics

Finally, exercise biomechanics is a critical area for study and research that provides valuable insights into how physical activity, exercise and sports affect the body and health. The principles of exercise biomechanics are rooted in the mechanics of human movement and the effects of physical activity and exercise on the body over time. The study of biomechanics allows us to optimize athletic performance and prevent injury-related disability. By understanding the mechanics of human movement, individuals can design effective exercise programs that target specific physical imbalances and impairments to improve their physical function and general well-being. Specifically, health-focused biomechanics is a specific and important research field to produce scientific evidence for overall health and well-being. Several methods are available for assessing biomechanical variables in different contexts related to health and well-being, each with its own advantages and limitations. The most effective method will depend on the specific information desired and the context in which the assessment is being conducted.

Exercise biomechanics is a complex and multidisciplinary field that provides valuable insights into how physical activity, exercise and sports affect body function and health. By understanding the principles of biomechanics, individuals can make informed decisions about their exercise program to optimize their physical health and well-being. Contributions in the form of research papers on sports biomechanics-related topics and their useful applications, as well as those discussing other topics of interest, such as physical activity, rehabilitation, physical fitness and competitive sport, will lead to improvement in the scientific knowledge and practices in this research field.
